# Supplementary figures and images for: All-trans retinoic acid changes muscle fiber type via increasing GADD34 dependent on MAPK signal
Source: Life Sci Alliance. 2022 Mar 22;5(7):e202101345. doi: 10.26508/lsa.202101345 (PMC8960774; doi:10.26508/lsa.202101345)

Fig 1B

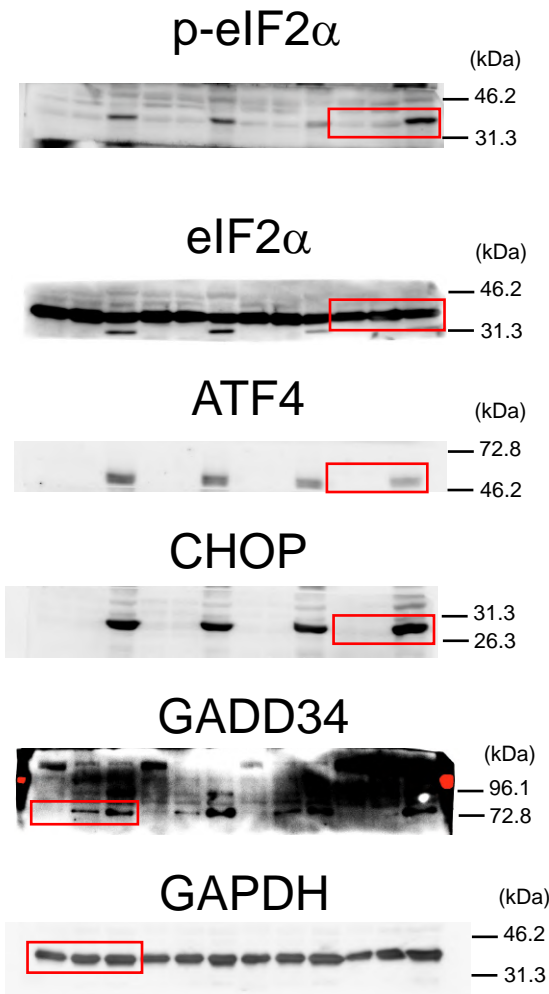

Fig 1E

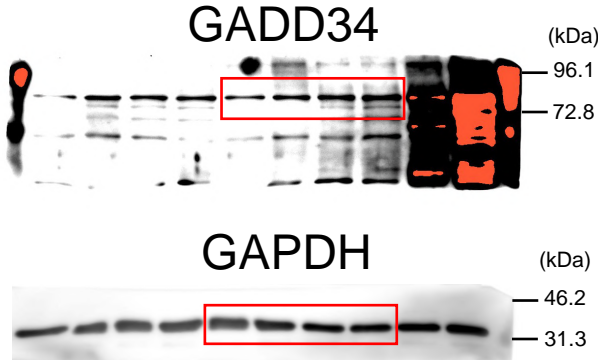

Supplement: Supplementary file 1 [file LSA-2021-01345_SdataF1.pdf]

Fig 2F

p-p38

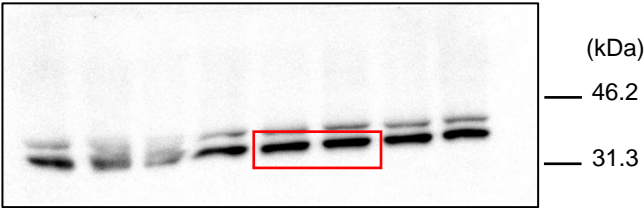

p38

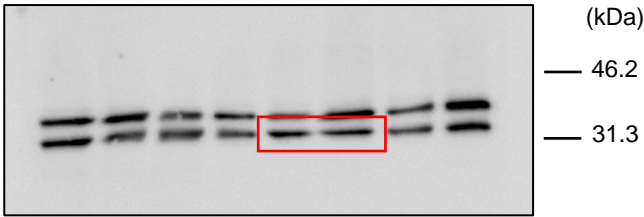

p-ERK

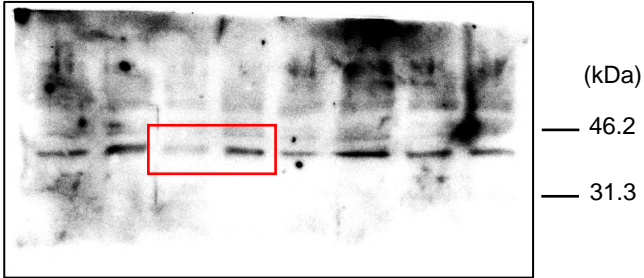

ERK

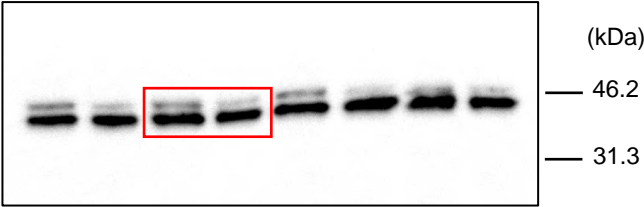

GAPDH

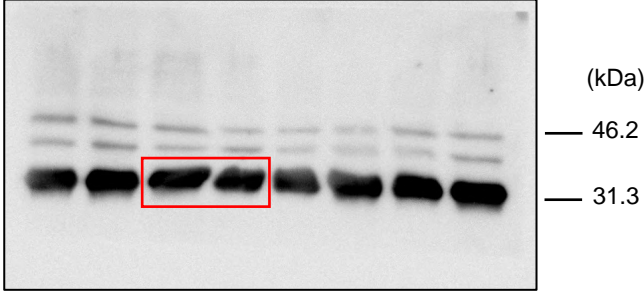

Fig 2G

p-p38

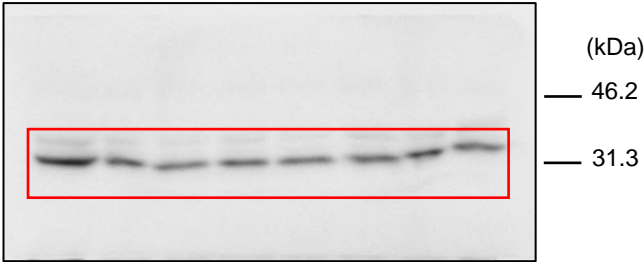

p38

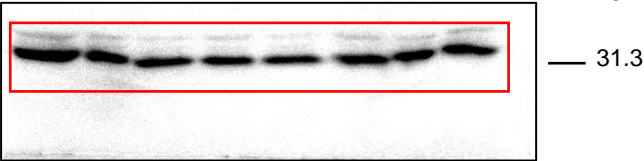

p-ERK

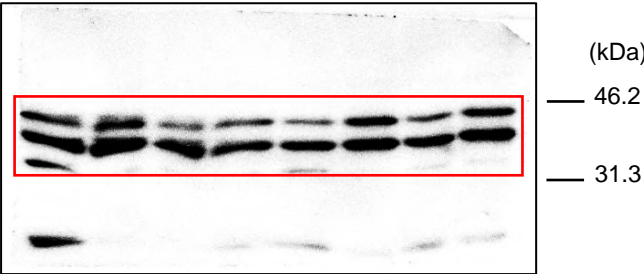

ERK

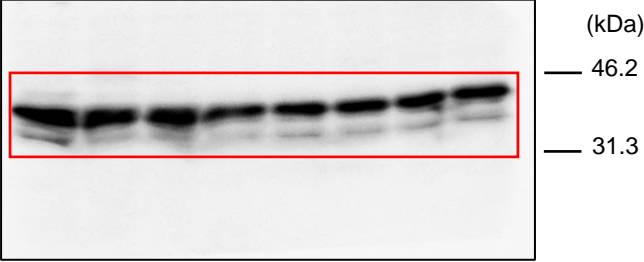

GAPDH

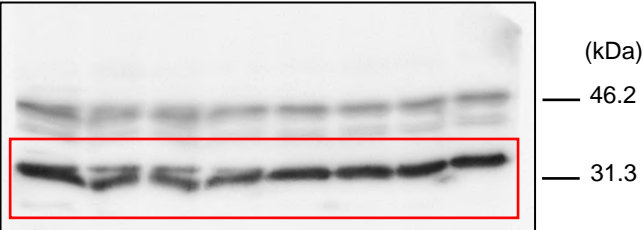

Supplement: Supplementary file 2 [file LSA-2021-01345_SdataF2.pdf]

Fig 3E

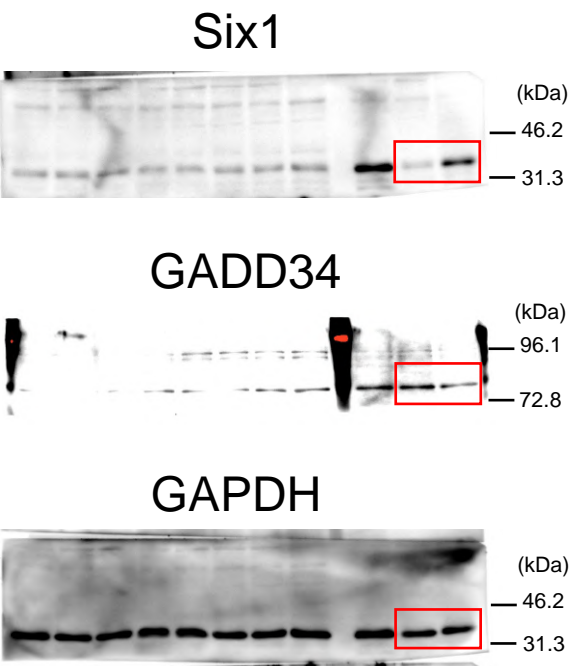

Fig 3I

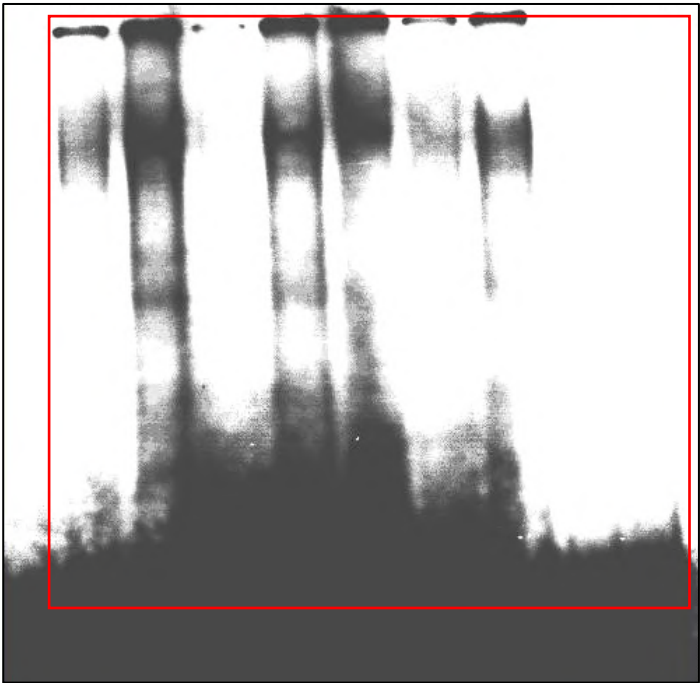

Supplement: Supplementary file 3 [file LSA-2021-01345_SdataF3.pdf]

Fig S2D

①

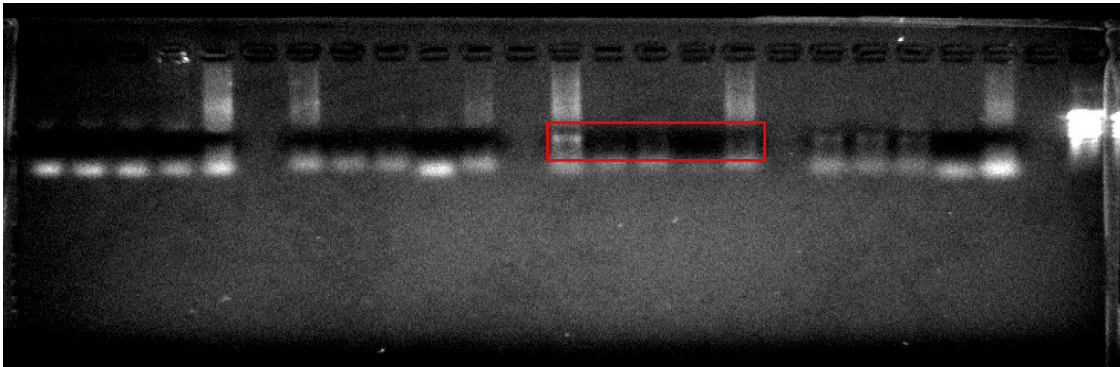

②

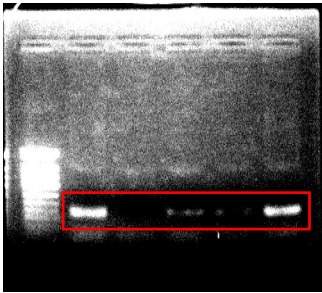

③

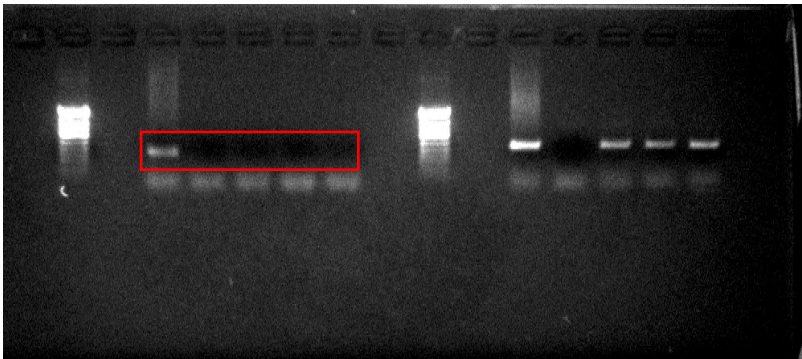

④

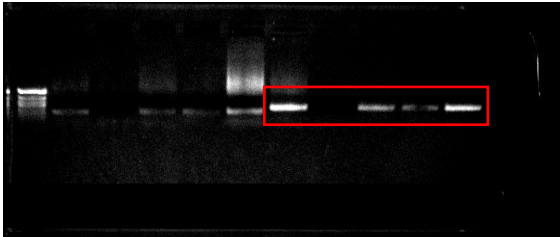

Supplement: Supplementary file 5 [file LSA-2021-01345_SdataFS2.pdf]

Fig 6A

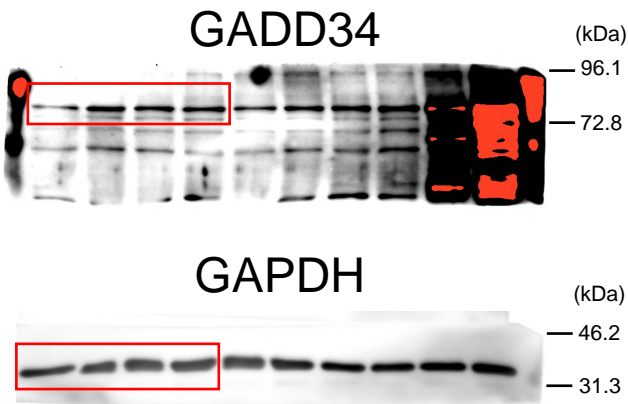

Fig 6G

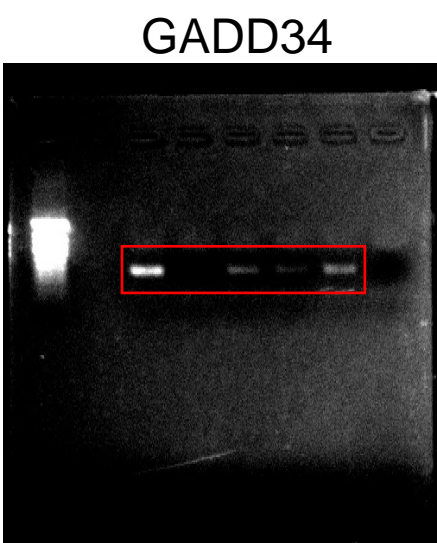

Supplement: Supplementary file 6 [file LSA-2021-01345_SdataF6.pdf]

Source data: Figure S3

Fig S3A

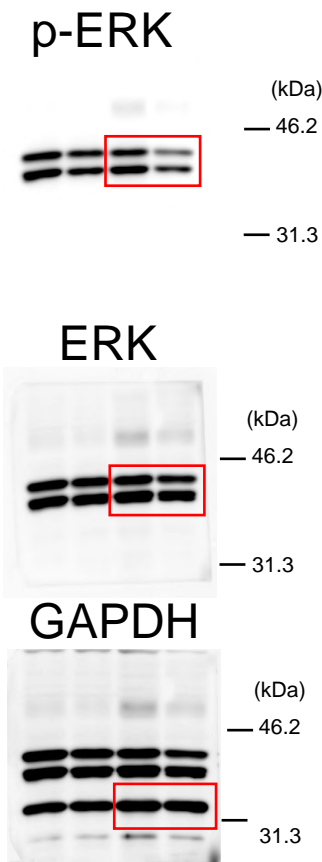

Fig S3B

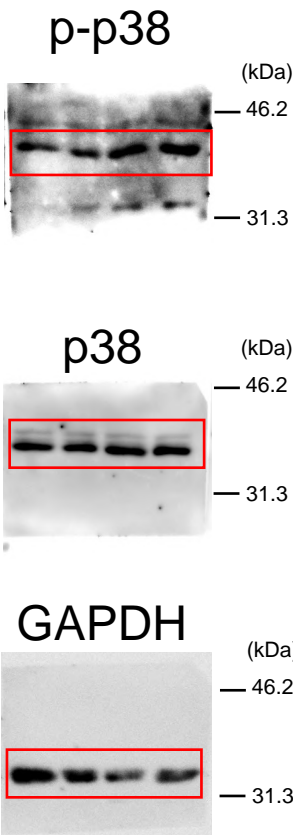

Fig S3C

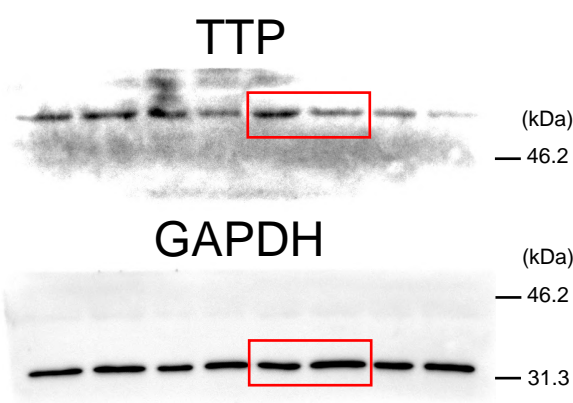

Fig S3D

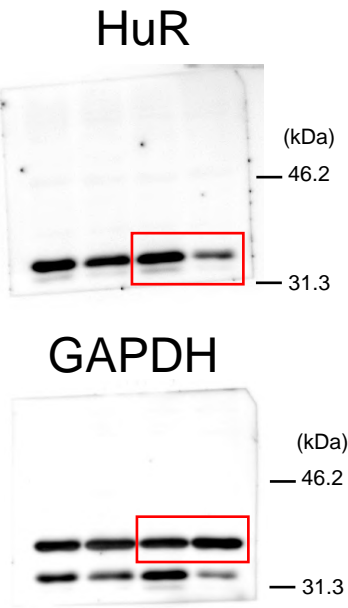

Supplement: Supplementary file 7 [file LSA-2021-01345_SdataFS3.pdf]

Fig S4D

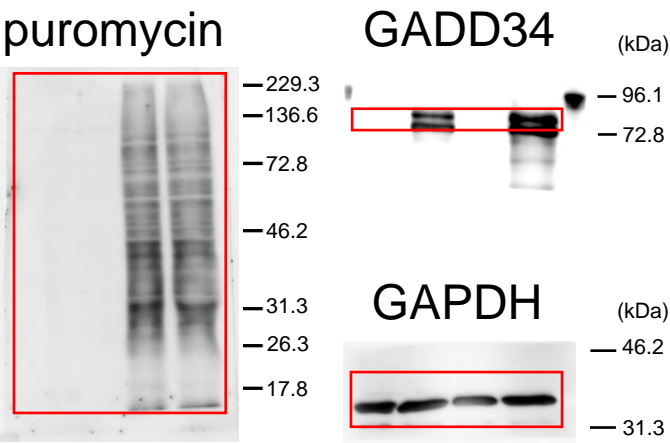

Supplement: Supplementary file 9 [file LSA-2021-01345_SdataFS4.pdf]
