# Supplementary material for: All-trans retinoic acid changes muscle fiber type via increasing GADD34 dependent on MAPK signal
Source: Life Sci Alliance. 2022 Mar 22;5(7):e202101345. doi: 10.26508/lsa.202101345 (PMC8960774; doi:10.26508/lsa.202101345)

Source data: Figure 4

Fig 4A

GADD34

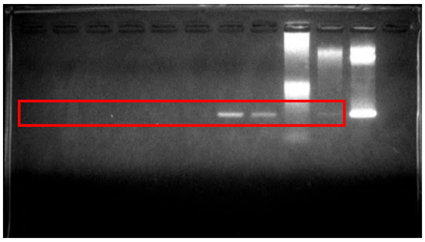

Ribosomal 18S

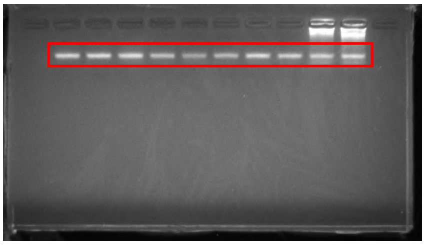

Fig 4B

Six1

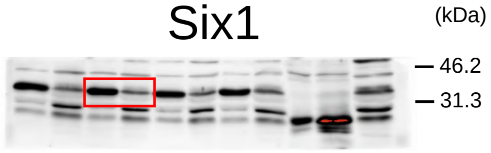

GAPDH

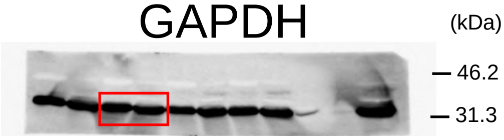

Fig 4C

Six1

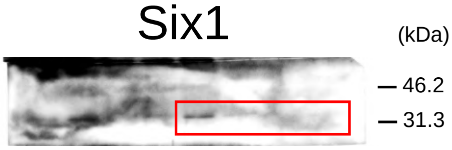

GAPDH

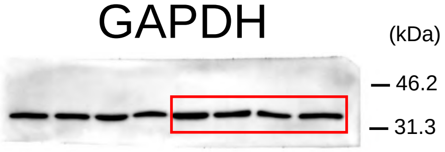

Fig 4F

Six1

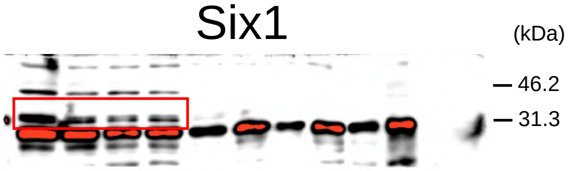

GADD34

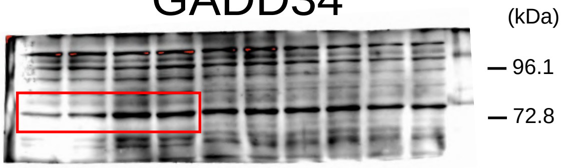

GAPDH

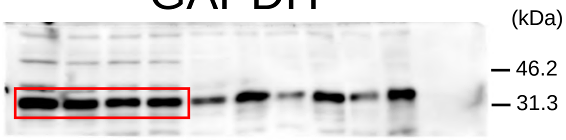

Fig 4H

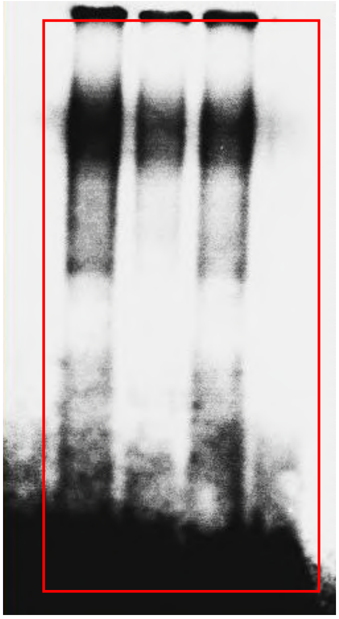

Supplement: Supplementary file 4 [file LSA-2021-01345_SdataF4.pdf]
