# Supplementary material for: All-trans retinoic acid changes muscle fiber type via increasing GADD34 dependent on MAPK signal
Source: Life Sci Alliance. 2022 Mar 22;5(7):e202101345. doi: 10.26508/lsa.202101345 (PMC8960774; doi:10.26508/lsa.202101345)

Source data: Figure 7-1

Fig 7B, C

empty DAY4 Cell-No.1

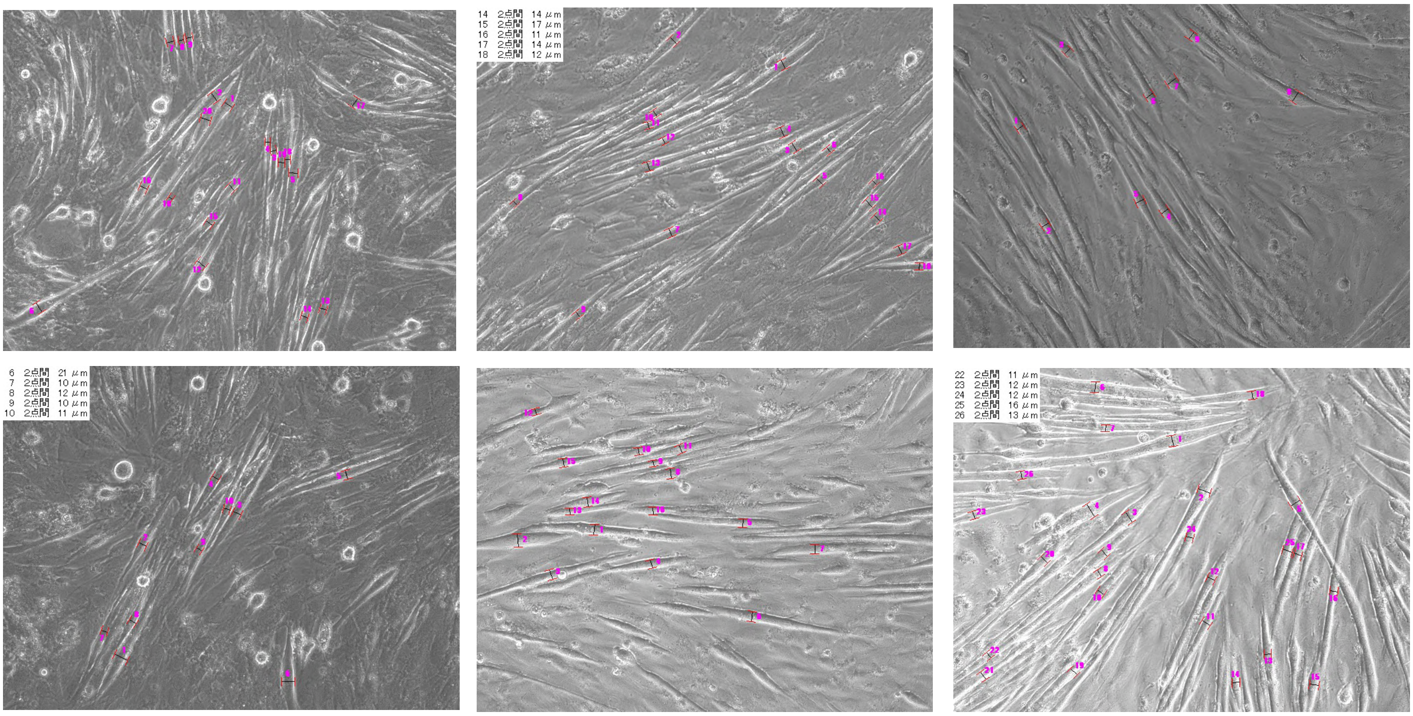

empty DAY4 Cell-No.2

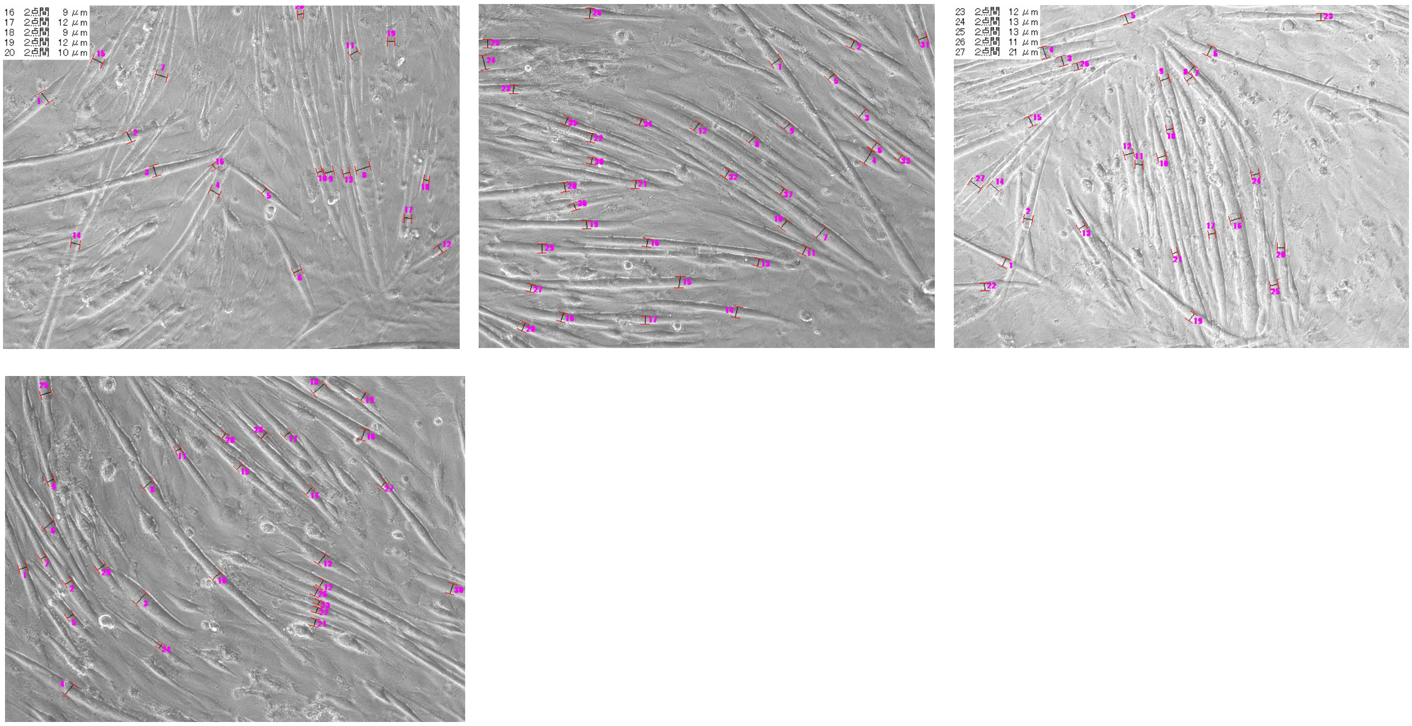

Source data: Figure 7-2

Fig 7B, C

empty DAY4 Cell-No.3

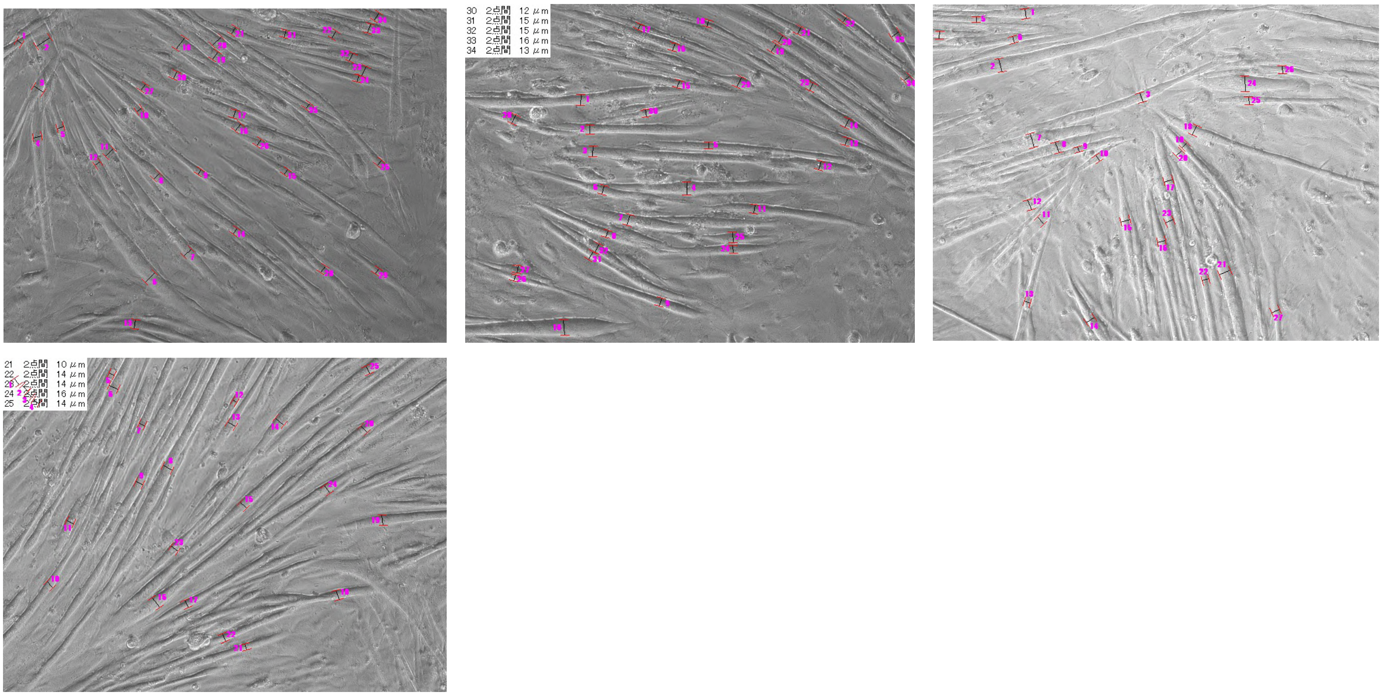

GADD34 DAY4 Cell-No.1

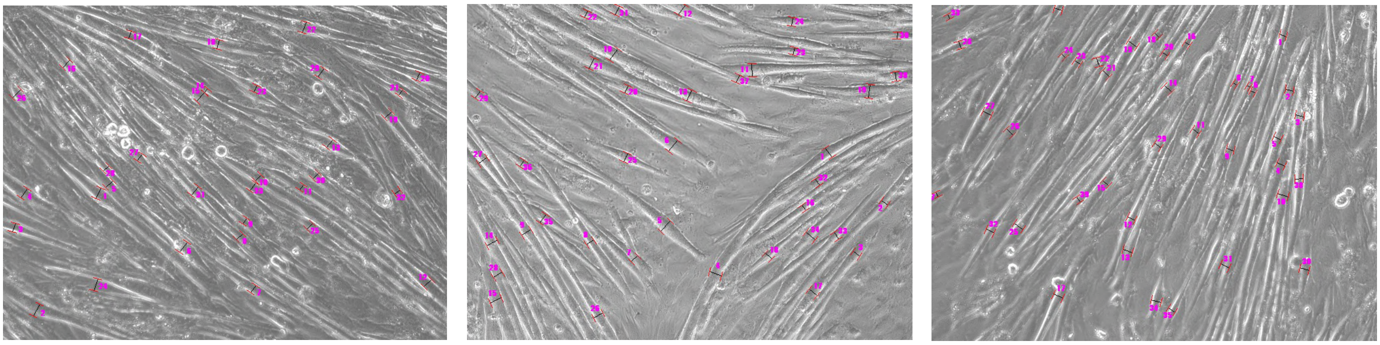

GADD34 DAY4 Cell-No.2

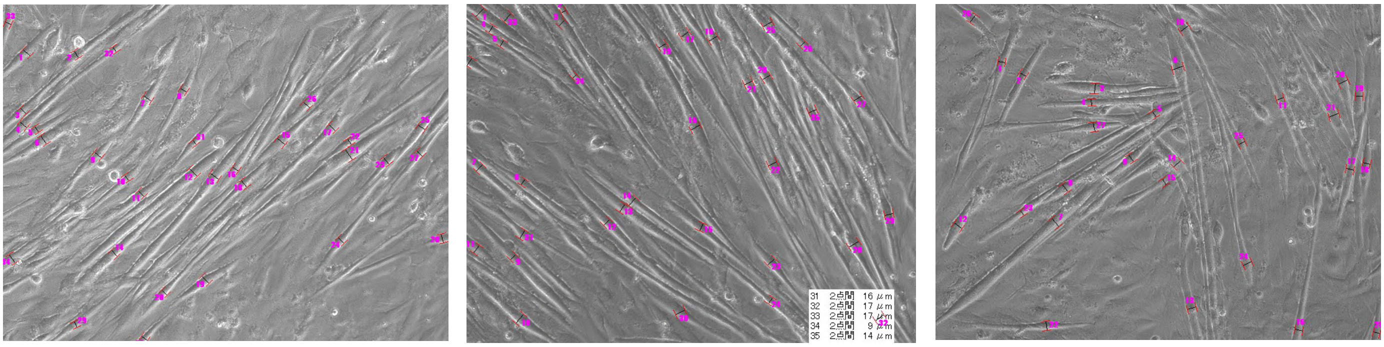

Source data: Figure 7-3

Fig 7B, C

GADD34 DAY4 Cell-No.3

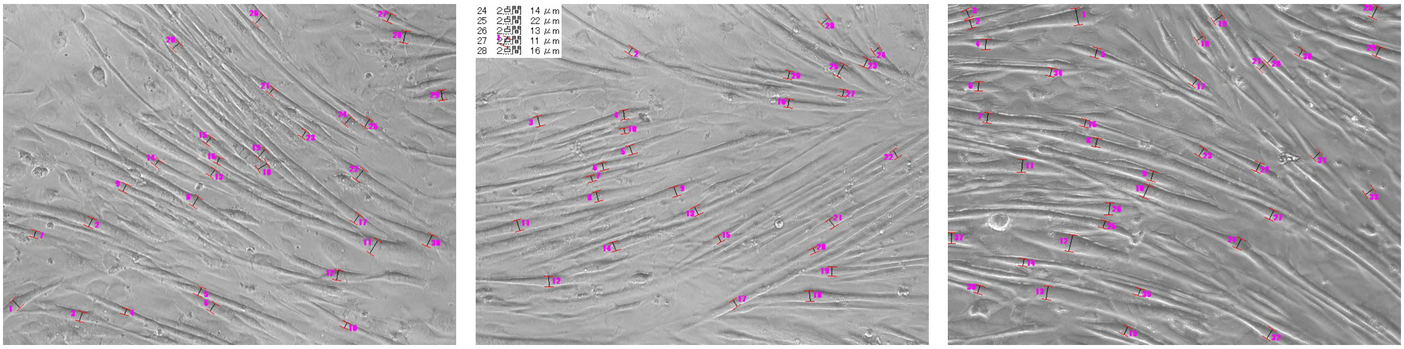

Fig 7D

Empty No.1

Nuclei: 607
MYHC positive nuclei: 104

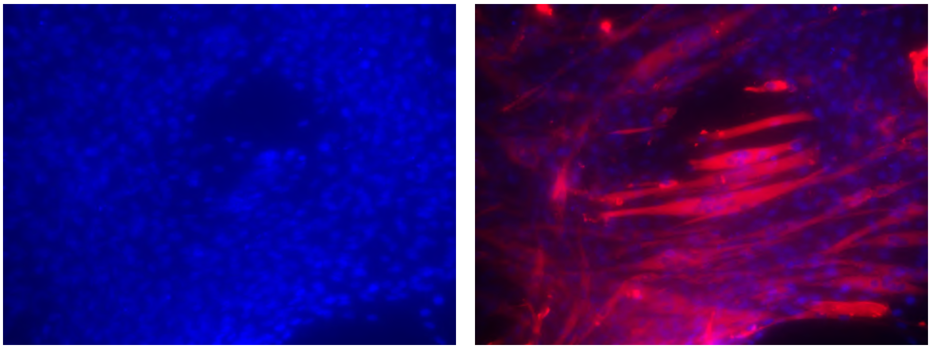

Empty No.2

Nuclei: 465
MYHC positive nuclei: 94

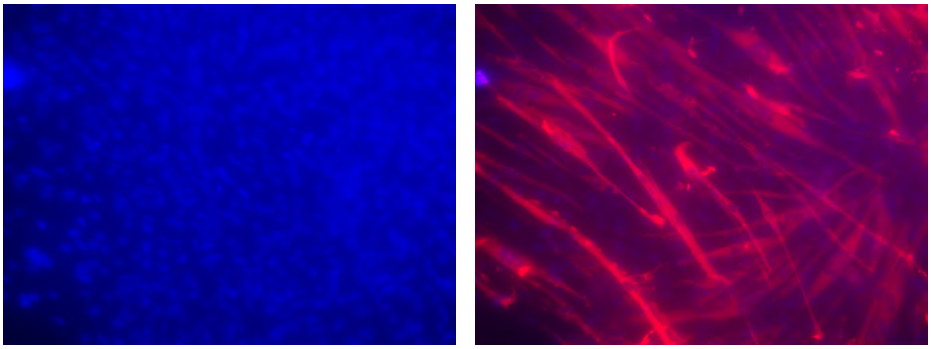

Empty No.3

Nuclei: 645
MYHC positive nuclei: 104

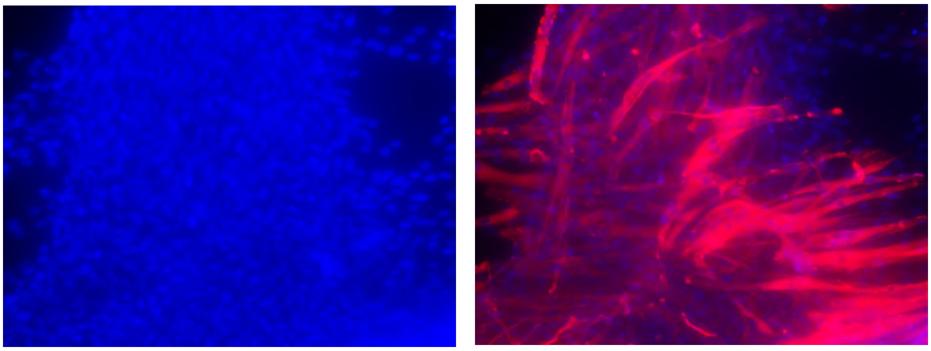

Source data: Figure 7-3

Fig 7D

Empty No.4
Nuclei: 662
MYHC positive nuclei: 102

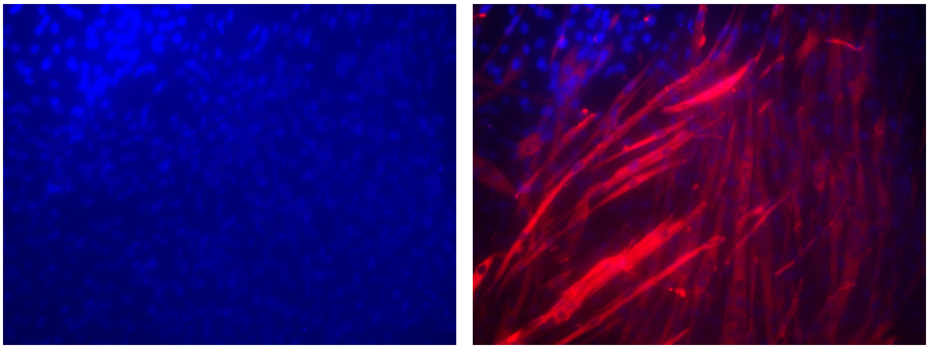

Empty No.5
Nuclei: 618
MYHC positive nuclei: 93

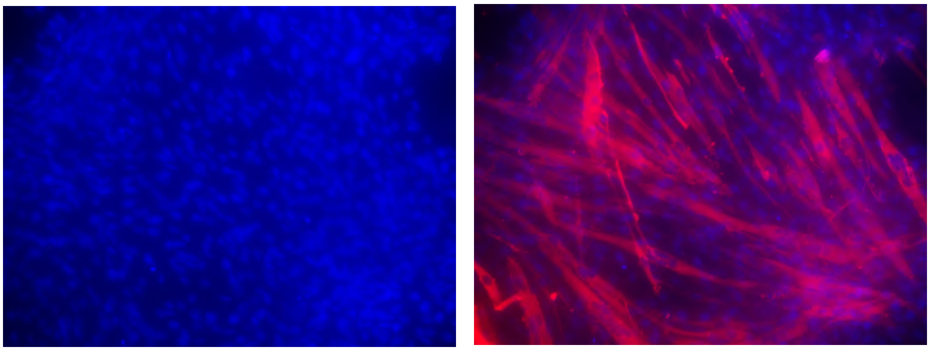

GADD34 No.1
Nuclei: 583
MYHC positive nuclei: 97

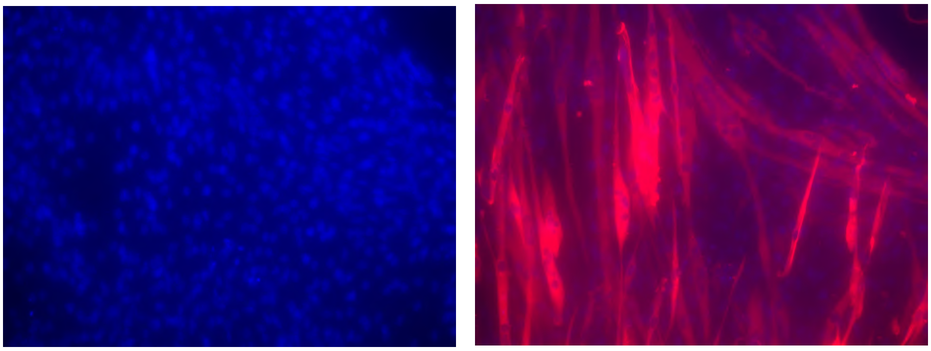

GADD34 No.2
Nuclei: 402
MYHC positive nuclei: 65

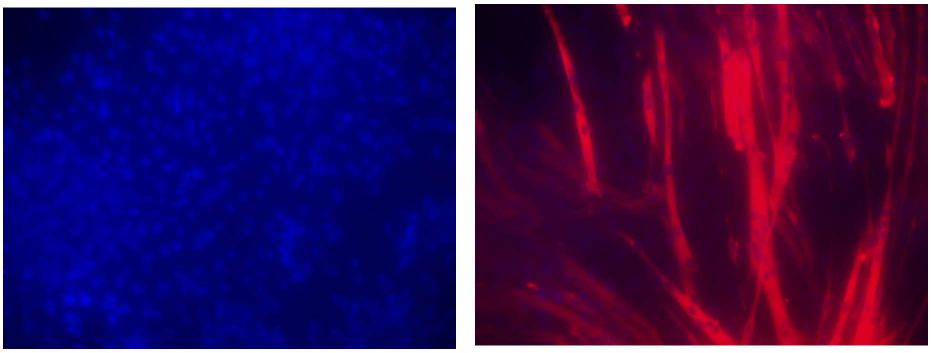

Source data: Figure 7-3

Fig 7D

GADD34 No.3

Nuclei: 625
MYHC positive nuclei: 98

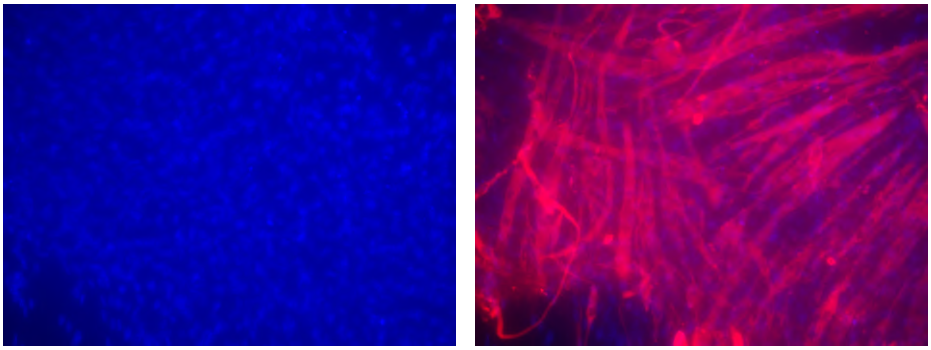

GADD34 No.4

Nuclei: 590
MYHC positive nuclei: 95

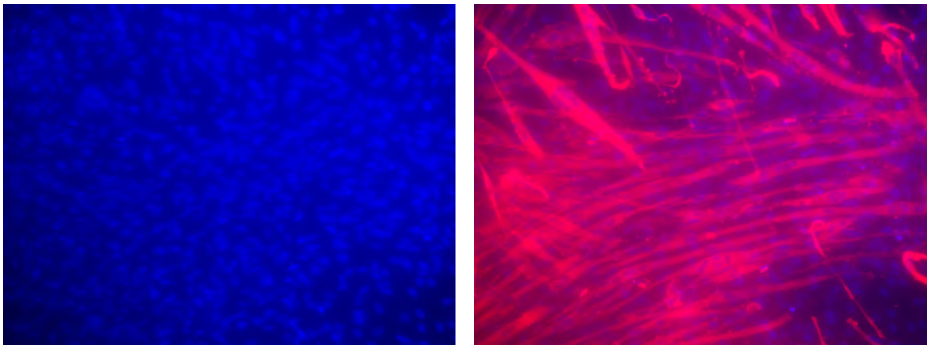

GADD34 No.5

Nuclei: 640
MYHC positive nuclei: 122

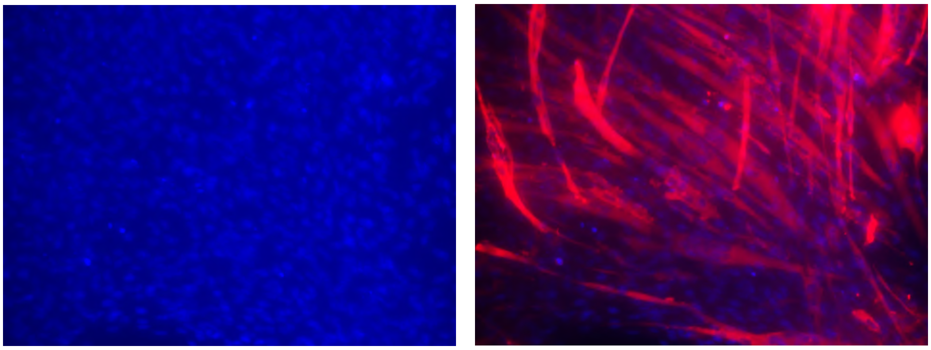

Source data: Figure 7-4

Fig 7E

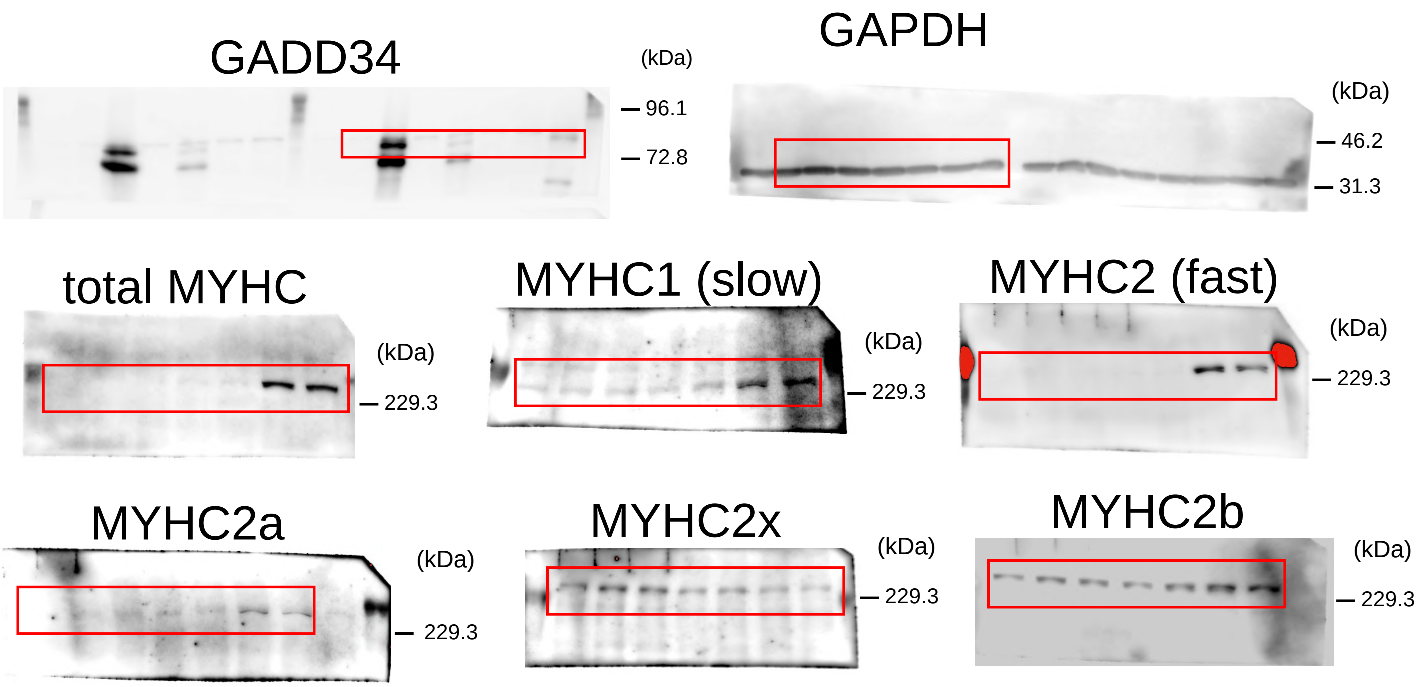

Fig 7G

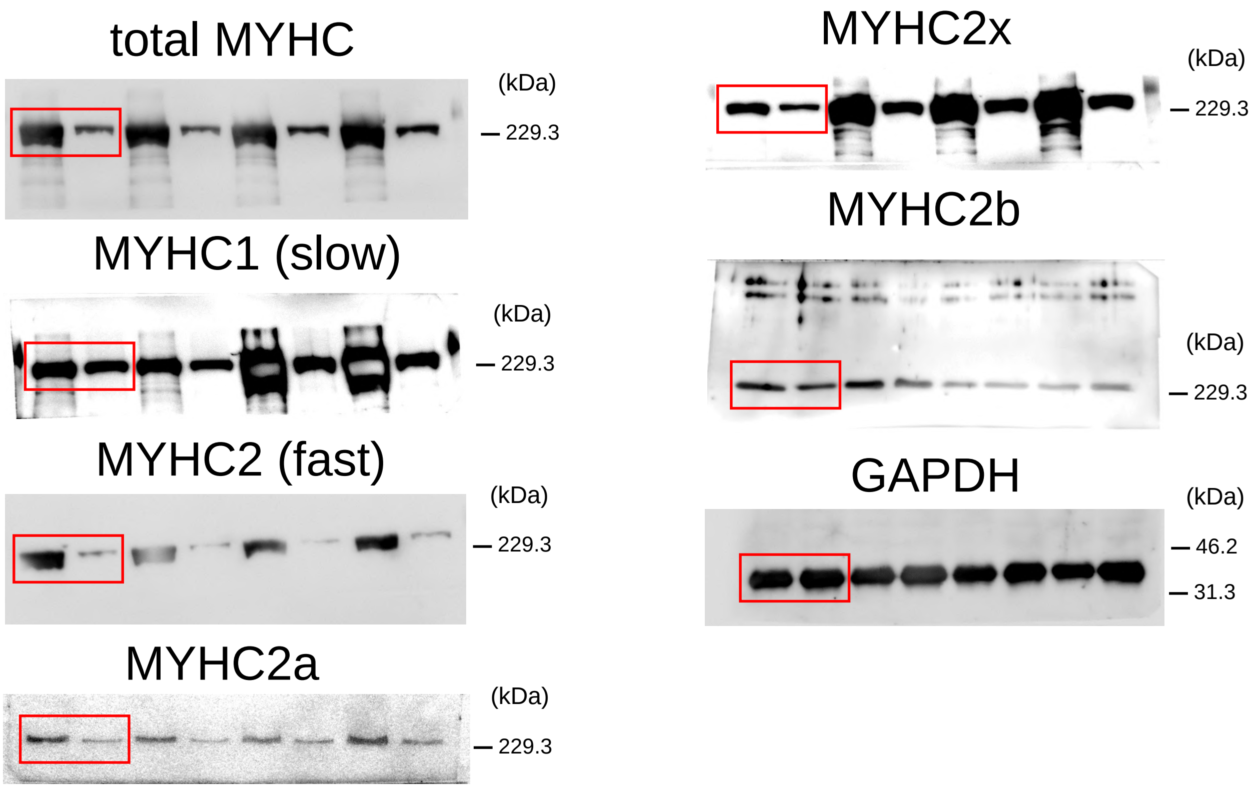

Supplement: Supplementary file 8 [file LSA-2021-01345_SdataF7.pdf]
